# Social and Health Disparities Among Relocated Public Housing Residents by Age: Implications for Aging in Place

**DOI:** 10.1093/geroni/igaa057.1564

**Published:** 2020-12-16

**Authors:** Nia Reed, Tiffany Young

**Affiliations:** 1 Georgia State University, Atlanta, Georgia, United States; 2 Lenell and Lillie Consulting, LLC, New Bern, North Carolina, United States

## Abstract

Research illustrates that neighborhood outcomes (including the built environment) influence the mental and physical health of vulnerable older adults (OA). Many OA aim to age-in-place but aging-in-place is less realistic for low-income OA because of gentrification and forced relocation. Examining neighborhood context is vital to understanding how the places we live contribute to well-being, yet, there is insufficient research on the biopsychosocial effects of forced relocation on low-income OA. To address this gap, our study uses aging-in-place theory to understand the association of neighborhood and health outcomes of relocated and nonrelocated low-income OA in public housing. This study includes three waves of data from Georgia State University’s Urban Health Initiative Study. Participants (n=225) were categorized by age (young-old = 50-64; old-old = 65-74; and oldest-old = 75+). We conducted multivariate regression analyses to highlight relationships between neighborhood and health outcomes, and relocation. We used geocoding to provide within-group analysis of relocated residents to determine if geographic proximity to former public housing communities affected neighborhood and health outcomes. Results show that relocated OA have worse informal social control and neighborhood satisfaction outcomes, but better built environment conditions than those who aged-in-place. Mental health and physical functioning worsened for relocated OA. Relocation was associated with reduced social cohesion and worse built environment conditions for the oldest-old. Moderation analysis illustrated that OA who relocated farther away had worse neighborhood outcomes. Considering the importance of aging-in-place to OA well-being, policymakers may reconsider forced relocation and allocating funds to enhance neighborhoods.

